# An emerging function of circRNA-miRNAs-mRNA axis in human diseases

**DOI:** 10.18632/oncotarget.19154

**Published:** 2017-07-10

**Authors:** Dawei Rong, Handong Sun, Zhouxiao Li, Shuheng Liu, Chaoxi Dong, Kai Fu, Weiwei Tang, Hongyong Cao

**Affiliations:** ^1^ Department of General Surgery, Nanjing First Hospital, Nanjing Medical University, Nanjing, Jiangsu, China; ^2^ Department of Neurosurgery, The First affiliated Hospital of Nanjing Medical University, Nanjing, Jiangsu, China; ^3^ Department of Oncology Surgery, Nanjing First Hospital, Nanjing Medical University, Nanjing, Jiangsu, China

**Keywords:** circular RNA, back-splicing, microRNA sponge, tumorgenesis

## Abstract

Circular RNAs (circRNAs), a novel class of long noncoding RNAs, are characterized by a covalently closed continuous loop without 5′ or 3′ polarities structure and have been widely found in thousands of lives including plants, animals and human beings. Utilizing the high-throughput RNA sequencing (RNA-seq) technology, recent findings have indicated thata great deal of circRNAs, which are endogenous, stable, widely expressed in mammalian cells, often exhibit cell type-specific, tissue-specific or developmental-stage-specific expression. Evidences are arising that some circRNAs might regulate microRNA (miRNA) function as microRNA sponges and play a significant role in transcriptional control. circRNAs associate with related miRNAs and the circRNA-miRNA axes are involved in a serious of disease pathways such as apoptosis, vascularization, invasion and metastasis. In this review, we generalize and analyse the aspects including synthesis, characteristics, classification, and several regulatory functions of circRNAs and highlight the association between circRNAs dysregulation by circRNA-miRNA-mRNA axis and sorts of diseases including cancer- related and non-cancer diseases.”

## INTRODUCTION

Circular RNAs (circRNAs) are a class of endogenous non-coding RNAs (ncRNAs) with a structure of covalently closed continuous loops, resulting in a more stable condition than line RNA [1]. In the early 1970s, the first circular RNA was found in some plant viroid and a circular form of RNA in the cytoplasm of monkey renal CV-1 cells was discovered via electro-microscopy by then [2–3]. With advances in high-throughput sequencing technology, recent studies have uncovered that a large number of circRNAs in human beings cells may be involved in human fetal development, myocardial Infarction as well as carcinomas [4–6]. In depth study revealed that some circRNAs might regulate microRNA (miRNA) function as microRNA sponges and the circRNA-miRNA-mRNA axis axes may play a crucial role in cancer-related or non-cancer pathways. For instance, Eur Heart J, et al confirmed that heart-related circRNA HRCR bound to miR-223 directly and suppressed expression of HRCR in human cardiomyocytes enforced attenuated hypertrophic responses [7]. Further studies indicated that circRNA have a potential value to provide novel strategies for clinical diagnostic and treatment of carcinomas [8]. In this review, we generalize synthesis, characteristics, classification, and several regulatory functions of circRNAs and highlight the association between circRNAs dysregulation and sorts of diseases including cancer-related and non-cancer diseases.

### Biogenesis of circular RNAs

**Figure 1:**
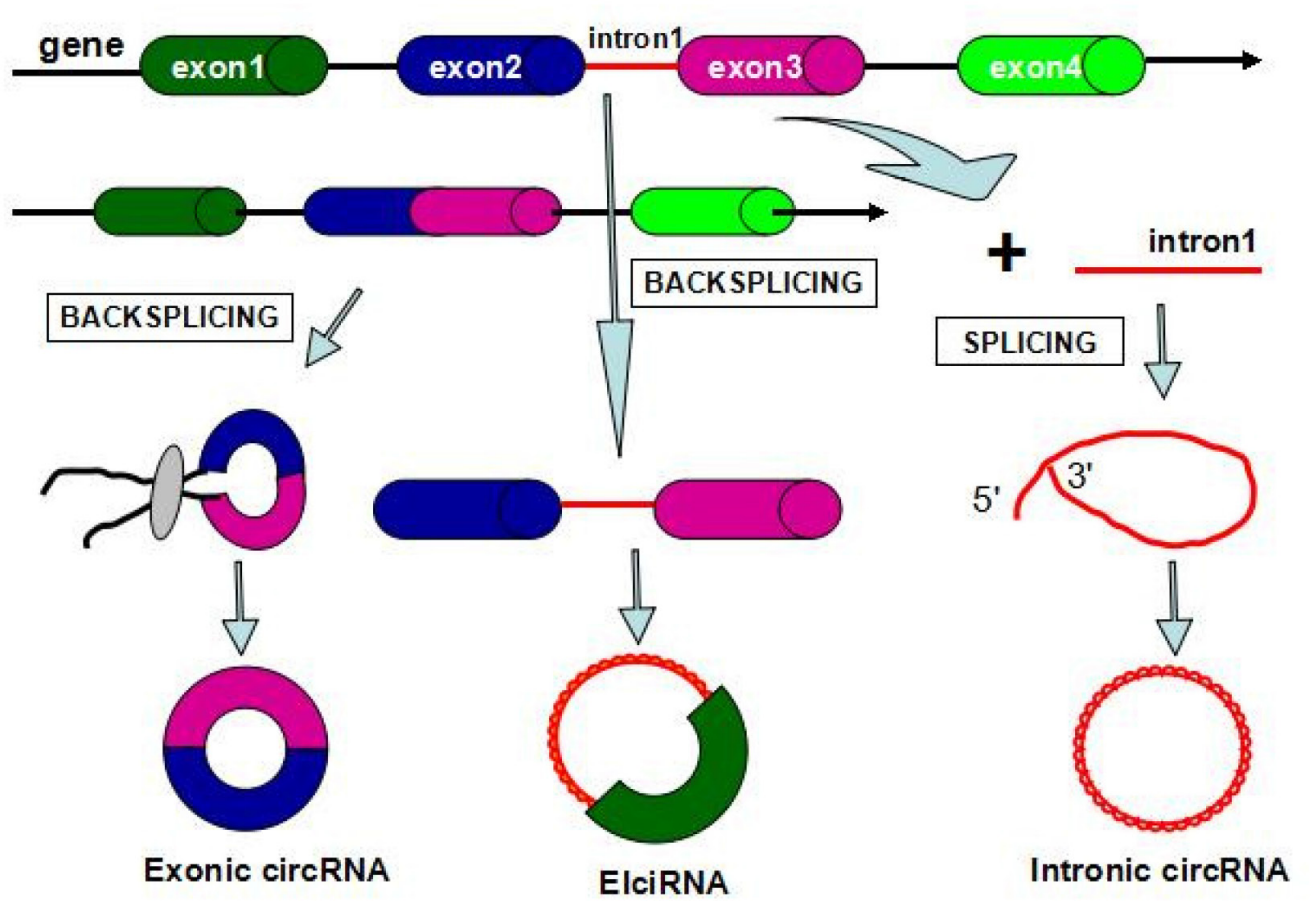
Biogenesis of circular RNAs in human diseases

Numerous studies have disclosed the biogenesis of circRNAs through back-splicing mechanism, which is different from the canonical splicing of long nocoding RNAs. circRNAs including exonic circRNAs, intronic circRNAs, and retained-intron circRNAs can be transcribed from pre-mRNAs sequences by RNA polymerase II (Figure 1) [9]. The distinctive structure of circRNAs contributes to a special form of splicing type: back-splicing that characterizes the synthesis of circular RNAs. During the non-canonical splicing process, RNA exons gain the circular form in a manner of head to tail and formed as exonic circular RNA, while the spliceosomal would cut out intronic genes in the binding part.

Previous researches have uncovered that exon circularization relies on flanking intronic complementary sequences and alternative formation of inverted repeated Alu pairs that could contribute to alternative circularization as well as resulting in multiple circular RNA transcripts produced from a single sequence [10]. The regulatory rules of circRNA biogenesis are dependent on the cis-regulatory elements and trans-acting factors which control splicing involved in the splicing process [9, 11]. Firstly, the biogenesis of circRNAs is regulated by a variety of RNA-binding proteins [14–15]. Meanwhile, the splicing factor Muscleblind (Mbl) promotes circRNA production from its own pre-mRNA [9]. Quaking (QKI), a type of RBP, are sufficient to bind to two flanking introns and strikingly bring the circularized exons closer together [12]. In addition, adenosine deaminase 1 acting on RNA (ADAR1) also participates in the regulation of synthesis of circRNAs by suppressing circRNA expression independently of its editing activity since it appears to function as an editing enzyme and a double-stranded RNA-binding protein [13]. The process of the back-splicing requires the canonical spliceosomal machinery and is associated with exon circularization efficiency, which is reliable on canonical splice sites bracketing the exons [9, 14–15]. As multiple circRNAs can be produced from the same gene sequences by alternative splicing, a large amount of circRNAs in mammals are processed from internal exons with long flanking introns, containing reverse complementary sequenceswhich are capable of pairing to form RNA duplexes [10].

Remarkablely, the low back-splicing efficiency is the characteristic of biogenesis of circRNA. This phenomenon possibly results from the presence of canonical splice sites where the exons and spliceosomes are detrimentally assembled at back-splicing sites to catalyze the ligation of upstream 3′ acceptor sites with downstream 5′ donor sites [9, 14–15]. Thus, the biogenesis of circRNAs is still unclear and remains to be further investigated.

### Characteristics of circular RNAs

With the development of bioinformatics technology and RNA sequencing, researchers have discovered thousands of circRNAs in most organisms including Archaea, human being and so on. Song X, et al detected >476 distinct circRNAs in human gliomas and Rybak-Wolf A, et al distinguished about 15, 849 distinct circRNAs in mouse and 65, 731 in human being. Moreover, Nair A, et al suggested that circRNA frequency in breast cancer may be useful for the diagnosis and treatment of breast cancer [16–18]. However, some circRNAs expressions are on the contrary, for example, circRNAs including Rims2, Tulp4 and Elf2 were found to be the major isoform of multiple genes in brain tissues [17].

The most important feature of endogenous circRNA is that the closed loop structures of circRNAs with a covalent bond linking the 3 ′ and 5 ′ ends make them avoid exonucleolytic degradation by RNase R [19]. For example, in an enzymatic process, circRNAs, known as Brolling circle amplification (RCA), play a main role in envolution of the development of hepatitis delta virus (HDV) and viroid replication models [20–21]. Noteworthy, some circRNAs are located in the nucleus in organism which harbors miRNA binding miRNA response elements and forming circRNA-miRNA axes are involved in regulation of gene expression at the transcription or post transcription level [10].

Base on the characteristic of circular RNAs, RNA-seq and high-throughput sequencing technology always are used to detect circRNAs. RNA-seq and high-throughput sequencing Technology, as the inevitable outcome of bioinformatics, have different adventage on detecting new circular RNA. Lot of circular RNA databases such as CircBase, CircNet, **CircInteractome** have been Established and could give more inforation about circRNA.

According to the recent studies, we could find that long noncoding RNAs and circular RNAs have some same aspects. The most meaningful point is that they all could function as a miRNA sponge and regulate downstream mRNA, all of which involved in the pathomechanism of various diseases including cancers. Meanwhile, they have different fields like that circular RNAs have stable structure and have more advantage to be a clinical biomarker than long noncoding RNAs, while long noncoding RNAs are superior to circular RNAs in abundance in human beings cells.

### Classification of circular RNAs

**Figure 2:**
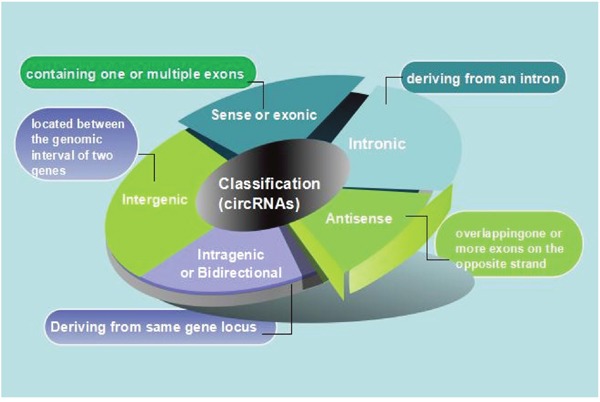
Diagrammatic drawing of circRNA classification **(a)** Sense or exonic, CircRNAs may contain just one or multiple exons which come from a linear transcript on the same strand, they show the probability for alternatively spliced isoforms. **(b)** Intronic, they may derive wholly from an intron of the linear transcript depend on a crucial motif which contain a 7-nt GU-rich element close to 11-nt C-rich element and an the 5splice site near the branch point. **(c)** Antisense, if it overlaps one or more exons of the linear sequence on the opposite strand. **(d)** Intragenic or Bidirectional, when circRNAs is transcribed from same gene locus of the linear sequence not classified as ‘sense’ and ‘intronic’ and whereas in close genomic proximity. **(e)** Intergenic, when it is located between the genomic interval of two genes.

The length of CircRNAs differs from each other and can derive from all of region of the genome [22–25]. Classification system of long noncoding RNAs (lncRNAs) could be used as an example to circular RNAs. According to their genomic proximity to the neighboring gene, circRNAs could be classified into five types: a. Sense or exonic, circRNAs may contain just one or multiple exons which come from a linear transcript on the same strand, they show the probability for alternatively spliced isoforms [10, 26]. b. Intronic, they may derive wholly from an intron of the linear transcript depend on a crucial motif which contain a 7-nt GU-rich element close to 11-nt C-rich element and an the 5splice site near the branch point [24]. c. Antisense, if it overlaps one or more exons of the linear sequence on the opposite strand. d. Intragenic or Bidirectional, when circRNAs is transcribed from same gene locus of the linear sequence not classified as ‘sense’ and ‘intronic’ and whereas in close genomic proximity. e. Intergenic, when it is located between the genomic intervals of two genes (Figure 2) [27].

### Function of circular RNAs

CircRNAs play a crucial role in gene regulation at the post-transcription or transcription level and then have an impact at the level of gene expression. In this review, several functions of circular RNAs were listed as follows.

### MiRNA sponges

**Figure 3:**
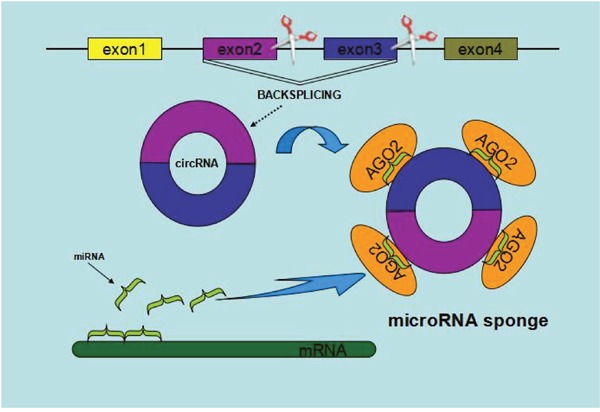
Schematic diagram of circRNAs functioning as miRNA sponges or competing endogenous RNAs

MiRNAs, an abundant class of small noncoding RNAs (∼22 nt), posttranscriptionally modulate the translation of target mRNAs via corresponding miRNA response elements [28]. The current studies have provided evidences that some of circRNAs harbor MREs, suggesting a potential role as competitive endogenous RNAs (ceRNAs) to compete for miRNA-binding sites, thus affecting miRNA activities [23, 29–30]. For example, CiRS-7, as a Human circRNA cerebellar degeneration-related protein 1 transcript (CDR1), acts as a miR-7 sponge by binding with related miRNA and influencing the availability of miR-7 to bind to its target mRNAs [30]. Besides ciRS-7, testis-specific circRNA, sex-determining region Y (circSRY), serves as the miR-138 sponge and functions as a platform for binding miR-138 and regulates linked mRNA translation [25, 30–32]. Additionally, Zheng Q, et al revealed an abundant circHIPK3 was observed to sponge to 9 miRNAs with 18 potential binding sites and in particular regulates cell growth by sponging multiple miR-124 and inhibiting miR-124 activity in malignant tumors [6]. Overall, the circ-miRNA axis, regardless of promotion or suppression, have significant effects on various pathways in human diseases and is worthy more thorough study (Figure 3) [33].

### Transcription regulation

Although most circRNAs regulate miRNAs as the role of miRNA sponge, some circular intronic RNAs (ciRNAs) or exon-intron circRNAs (EIciRNAs) are predicted to function as post-transcriptional regulators of gene expression and regulate linear RNA transcription and protein expression [22, 34–35]. Some ciRNAs such as c-sirt7, derived from lariats and interacting with the Pol II complex make the transcription levels of the relevant ankyrin repeat domain 52 (ANKRD52) or sirtuin 7 (SIRT7) genes decline. All together, abundant ciRNAs promote transcription of their parental genes with the unknown mechanism [24]. Meanwhile, EIciRNAs mainly localized in the nucleus, connect with U1 snRNP and ascend transcription of related parental genes [36].

### Competition with linear splicing

Back-splicing as circRNA biogenesis itself has been suggested that the usage of 5′ and 3′splice sites can compete with pre-mRNA splicing contribute to lower levels of linear mRNAs that include the exons circularRNAs [9–10]. An circularized exon means that it will not be involved in the processed mRNA and occurs in expense of linear splicing to produce mRNAs [37]. therefore, the synthesis of back-spliced circRNAs lead to the consequence of the downregulation of protein coding mRNAs. Future work revealed a large proportion of genes could take part in generating circRNAs and the process of circRNA biogenesis is happening along with the whole life. alternative splicing mechanisms like a switch who make the decision to back-splice or splice linearly. Consequently, a crucial gene regulatory mechanism involving this competition could be established and get more attention [22, 35].

### Other functions

Increasing evidences from the studies of circ-Foxo3 uncover that circ-Foxo3 can sequester proteins in the cytoplasm and prevent their nuclear entry by binding these proteins in the cytoplasm. On the contrary, knockdown of circ-Foxo3 which interacting with stress-related proteins FAK and HIF-1α may enhance nuclear expression of these proteins [22, 38–39]. Moreover, circRNAs with open reading frames also may be translated into peptides. For example, Haidar et al. found a circRNA of the virusoid in rice yellow mottle virus can code for a protein [40].

### Association between circRNA-miRNA axis and progression of human diseases

CircRNAs have been reported to be involved in many human diseases especially for carcinomas. To date, several studies suggest that the circRNA-miRNA-mRNA axis axis is probably involved in signaling pathway of human diseases by regulating pathogenicity-related gene expression.

### Circular RNAs in non-cancer diseases

In this review, we listed the expression of circRNAs and circRNA-miRNA axis in signaling pathway of various types of non-cancer diseases such as major depressive disorder, myocardial infarction and provide potential implications in targeted therapy (Table 1).

**Table 1:** Summary of the expression of circRNAs and circRNA-miRNA axis in signaling pathway of non-cancer diseases

Type of non-tumor diseases	Sequence name or more content	Expression	Intersection molecules and/or pathway	First author
TLR4/LPS	circ-RasGEF1B	↑	circ-RasGEF1B↓-ICAM-1↓-protecting cells against microbial infection	Ng, W. L [41].
Myocardial Infarction	CircRNA Cdr1	↑	CircRNA Cdr1↑-miR-7a↓-PARP, SP1-Myocardial Infarction	Geng, H.H. [42].
osteoarthritis	Circ-CER	↓	CircRNA-CER/MMP13-miR-136- chondrocyte ECM degradation	Liu, Q. [43].
cardiac senescence	circ-Foxo3	↑	circ-Foxo3↑-ID-1↓, E2F1↓, FAK↓, HIF1alpha↓-cardiac senescence	Du, W. W [38].
heart failure	circ-HRCR	↓	circ-HRCR↑- miR-223-ARC-reduced hypertrophic responses	Wang, K [7].
immunosenescence	circRNA_100783	↑	circRNA100783-miR-mRNA-CD28-related CD8(+) T cell ageing	Wang, Y. H [44].
diabetes	Cdr1as	↑	long-term forskolin/PMA-Cdr1as↑-miR7-cAMP-PKC-Pax6/Myrip	Xu, H. [45].
brain tissue	ciRS-7	↑	ciRS-7 - miR-7(Ago)	Hansen, T. B [30].
	Circ-Sry	↑	Sry-miR-138	
lead-induced neuronal cell apoptosis	circ-Rar1	unknown	circRar1-miR-671- apoptosis-associated caspase8/p38	Nan, A. [46].
nonalcoholic steatohepatitis	circRNA_002581	unknown	circRNA_002581-miR-122-Slc1a5/Plp2/Cpeb1	Jin, X. [47].
	circRNA_007585	unknown	circRNA_007585-miR-326- UCP2	
Hirschsprung's disease	Circ-ZNF609	↓	ZNF609- miR-150-5p - AKT3-proliferation, migration	Peng, L. [48].
cardiac fibroblasts	CircRNA_000203	↑	CircRNA_000203↑-miR-26b-5p↓- Col1a2 and CTGF	Tang, C. M. [49].

↓ means down-regulated.

↑ means up-regulated.

Some circRNAs present as an upward trend to regulate the pathways. Circ-Foxo3, originated from one of the forkhead family of transcription factors Foxo3, could both act as miRNA sponges for miR-136, miR-138, miR-433, miR-762, miR-3614-5p and influencing their function [50]. Du WW, et al tested the roles of a circular RNA circ-Foxo3 in senescence both *in vitro* and *in vivo* and found that circ-Foxo3 was highly expressed in heart of aged patients and mice and it binded to the anti-senescent protein ID-1 and the transcription factor E2F1 and the anti-stress proteins FAK and HIF1α. On the contrary, they also found that silencing endogenous circ-Foxo3 suppressed senescence of mouse embryonic fibroblasts and ectopic expression of circ-Foxo3 relieved senescence [38].

Meanwhile, some other circRNAs play a downward regulation in non-cancer related diseases. For instance, Liu Q. et al discovered that circRNA-CER decreased expression in osteoarthritis (OA) and regulated MMP13 expression as a competing endogenous RNA (ceRNA) and involving in the processof chondrocyte ECM degradation, suggesting that circRNA-CER could be used as a potential target in OA therapy [43]. Peng L, et al reported that the cir-ZNF609 was down-regulated in Hirschsprung disease (HSCR) compared with normal adjacent tissues and may also act as a sponge for miR-150-5p to regulate the expression of AKT3 which is linked with the proliferation and migration of cells. Furthermore, silencing cir-ZNF609 inhibited the migration and proliferation of cells. In general, these findings expounded that cir-ZNF609 participate in the onset of HSCR through the cir-ZNF609-miR-150-5p-AKT3 axis [48].

### Circular RNAs in carcinomas

We also offered the table about the expression of circRNAs and circRNA-miRNA axis in signaling pathway of some sorts of human cancers such as neuroglioma, colorectal cancer (Table 2).

**Table 2:** Summary of the expression of circRNAs and circRNA-miRNA axis in signaling pathway of cancers

Type of cancer	Sequence name or more content	Expression	Intersection molecules and/or pathway	First author
bladder carcinoma	circ-TCF25	↑	circTCF25↑-miR-103a-3p↓/miR-107↓-CDK6↑-proliferation, migration↑	Zhong, Z. [50].
esophageal cancer	CircRNA_001059	↑	CircRNA_001059-miRNA (Wnt pathway)	Su, H. [51].
	CircRNA_000167	↓	CircRNA_000167-miRNA (Wnt pathway)	
hepatoma carcinoma	CircRNA Cdr1	↑	CircRNA Cdr1↑-miR-7↓-CCNE1, PIK3CD↓-proliferation, invision↑	Yu, L. [52].
bladder carcinoma	Circ-MYLK	unknown	CircRNA MYLK-miR-29a-3p-DNMT3B	Huang, M. [53].
hepatoma carcinoma	hsa_circ_0005075	unknown	hsa_circ_0005075-miR-23B-5P/93-3P/581/23A-5P-HCC tumor size	Shang, X. [54].
Colorectal Cancer	hsa_circ_001569	↑	circRNA_001569-miR-145↓-E2F5, BAG4, FMNL2↑-proliferation, invision	Xie, H. [55].
seven cancers	circ-HIPK3	↓	circHIPK3-miR-124-cell growth	Zheng, Q. [6].
neuroglioma	circRNA	unknown	miR-671-5p↑/CDR1-AS↓/CDR1↓/VSNL1↓-migration↑, migration↑	Barbagallo, D [56].
carcinoma	circ-Foxo3	↓	circ-Foxo3↑--p21-CDK2↓-cell cycle progression↓	Du, W. W [57].
Colorectal Cancer	cir-ITCH	↓	cir-ITCH↓-ITCH↑-Wnt/beta-catenin↓	Huang, G [58].
esophageal carcinoma	cir-ITCH	↓	cir-ITCH↓-miR7/17/214-ITCH↑-phosphorylated DV12-Wnt/beta-catenin↓	Li, F [59].
breast cancer	circ-Foxo3	↑	Foxo3P↑-Foxo3 circ/Foxo3 mRNA-proliferation↓	Yang, W. [8].
carcinoma	circ-CDR1	unknown	CDR1↓-miR-671-CDR1 mRNA ↓	Hansen, T. B [60].
neuroglioma	cZNF292	↓	cZNF292↓-Wnt/beta-catenin signaling pathway-PRR11, Cyclin A, p-CDK2, VEGFR-1/2, p-VEGFR-1/2, EGFR-proliferation, cell cycle progression↓	Yang, P. [61].
hepatoma carcinoma	ciRS-7 (Cdr1as)	↓	ciRS-7-miR-7-PIK3CD/p70S6K	Xu, L. [62].
lung cancer	circ-ITCH	↓	ITCH-miR-7/miR-214 (Wnt/beta-catenin signaling pathway) -A549/NIC-H460	Wan, L. [63].
carcinoma	circ-Foxo3	↓	circ-Foxo3↑- Foxo3↑, p53↓- tumor apoptosis	Du, W. W. [64].
KRAS mutant colon cancer	circRNA	↓	mutant KRAS- circRNA abundance↓	Dou, Y. [65].
clear cell renal cell carcinoma	circ-HIAT1	↓	AR - circHIAT1↓-miR-195-5P/29a-3p/29c-3p↓-CDC42↑-CCRCC migration and invasion	Wang, Kefeng [66].

↓ means down-regulated.

↑ means up-regulated.

Some circRNAs, participating in the interaction with miRNAs, play a role as a upward trend to regulate the pathways significantly in human cancers. Zhong et, al reported that circTCF25 binded to miR-103a-3p/miR-107 and potentially contributed to the up-regulation of thirteen targets about cell proliferation, migration and invasion. Subsequently, the phenomena that down-regulating miR-103a-3p and miR-107, increasing CDK6 expression, and promoting proliferation and migration were observed by over-expression of circTCF25. All of these suggested that circTCF25 could be involved in pathway of bladder carcinoma via circTCF25-miR-103a-3p/miR-107-CDK6 axis and be a new potential marker for this cancer [50].

We also pay attention to some circRNAs that playing a downward regulation in carcinomas. Up to date, several evidence has demonstrated that androgen receptor (AR) is related with promoting the mechanisms about metastasis of clear cell renalcell carcinoma (ccRCC). Wang K et al identified a new circRNA (named as circHIAT1) with lower expression in ccRCCs, compareing with adjacent nomal tissues. AR-suppressed circHIAT1 suppress ccRCC cell progression by rising circ HIAT1 expression. In this newly signal process, circHIAT1 could deregulate miR-195-5p /29a-3p/29c-3p axis and increase CDC42 expression to fulfil the goal of enhancing ccRCC cell migration and invasion. Conclusively, they suggested that AR-circHIAT1 -mediated miR-195-5p/29a-3p/29c-3p/CDC42 axis may give us novel insights into develop new therapies to cure ccRCC [66]. Yang P et al reported cZNF292 is an important circular oncogenic RNA and plays a critical role in tube formation. According to the information that the Wnt/β-catenin signaling pathway and related genes such as Cyclin A, p-CDK2, VEGFR-1/2 and EGFR were involved in the process of halting the Cell cycle progression in humanglioma U87MG and U251 cells. they found that cZNF292 silencing plays an critical role in suppressing tube formation by inhibiting glioma cell proliferation and cell cycle progression and may serve as new potential targets and biomarkers for glioma therapy [61].

### Conclusion and perspective

CircRNAs, a recently discovered RNA type, were thought to be the products of transcription errors. With the development of high-throughput sequencing technologies and bioinformatics, they have become a popular research subject and gain more attention currently. circRNAs as miRNA sponge specifically bind to microRNAs and then modulate gene expression by competing with competing endogenous RNA, providing a more novel understanding of the RNA language and their role in signaling pathway of human diseases. CircRNAs are expressed widely in tissues, saliva, blood, exosomes and much more stable owning to their circular structure. According to these characteristics, circRNAs could be as biological markers of human diseases and thus improve the accuracy and specificity of diagnosis and therapies.

In addition, the understanding and achievements in the study of circRNAs are still very limited at present. Numerous problems about circular RNA are unknown. For example, with the natural stable structure, how circRNAs are degraded rightly in final? Are there any other molecular mechanism of synthesis method of circRNAs? The meaning and value of circRNA is obvious and it is crucial to do more research on circRNAs, especially the circRNA biogenesis.
